# Efficacy of Empirical Antibiotics in Community-Based Management of Uncomplicated Severe Acute Malnutrition in Children: Protocol of a Randomized Controlled Trial

**DOI:** 10.2196/91579

**Published:** 2026-09-02

**Authors:** Gomathi Ramaswamy, Sirisha Cheela, Kishore Yadav Jothula, Mounika Reddy, Raja S, Anand K Pyati, Patil Parag Parushuram, Priyanka Kotni, Khyati Tiwari, Sangeetha Sampath, Rahul Narang

**Affiliations:** 1 Department of Community Medicine and Family Medicine All India Institute of Medical Sciences Bibinagar Yadadri Bhuvanagiri, Telangana India; 2 Department of Pediatrics All India Institute of Medical Sciences Bibinagar Yadadri Bhuvanagiri, Telangana India; 3 Department of Microbiology All India Institute of Medical Sciences Bibinagar Yadadri Bhuvanagiri, Telangana India; 4 Department of Biochemistry All India Institute of Medical Sciences Bibinagar Yadadri Bhuvanagiri, Telangana India; 5 Department of Pathology All India Institute of Medical Sciences Bibinagar Yadadri Bhuvanagiri, Telangana India; 6 United Nations Children's Fund India Banjara Hills, Hyderabad, Telangana India

**Keywords:** severe acute malnutrition, Community-based Management of Acute Malnutrition, empirical antibiotics, antimicrobial resistance, randomized controlled trial

## Abstract

**Background:**

An estimated 17 million children have severe acute malnutrition (SAM) worldwide, with the majority residing in South Asia. Routine empirical antibiotics are prescribed for Community-based Management of Severe Acute Malnutrition (CSAM) to address presumed clinical/subclinical infections and improve nutritional recovery for children with uncomplicated SAM, but low prevalence of infection has been reported. Thus, targeted antibiotics (eg, single-dose azithromycin or 7-day amoxicillin) are recommended over empirical antibiotics to limit emerging antimicrobial resistance (AMR), but there is limited literature on the relevance.

**Objective:**

This study aims to (1) estimate the prevalence of clinical/subclinical infections and describe AMR patterns among children aged 6-59 months with uncomplicated SAM and assess associations with sociodemographic, clinical, and nutritional factors; (2) explore health-seeking behavior, antibiotic use, and care preferences among caregivers and health care providers; and (3) evaluate the efficacy of 7-day amoxicillin versus single-dose azithromycin versus no antibiotics in weight gain and nutritional recovery among children with SAM.

**Methods:**

This mixed methods study will be part of the ongoing CSAM program implemented in selected districts of Telangana, India. Phase 1 will be a cross-sectional analytical study with a laboratory component to estimate the prevalence of clinical/subclinical infections and AMR among 606 children. Phase 2 will use a qualitative design (focus group discussions with caregivers and in-depth interviews with health care providers) to explore perceptions and practices related to management and antibiotic use among the children. Phase 3 will be an open-label, parallel-group randomized controlled trial (RCT) with three groups of ~250 children each to compare nutritional recovery: 7 days of amoxicillin + standard nutritional care, single-dose azithromycin + standard nutritional care, and no antibiotics + standard nutritional care (control). Children will be followed for up to 6 months, with periodic anthropometric, clinical, and laboratory assessments.

**Results:**

Participant recruitment began in June 2026. Baseline assessments, interventions, and final evaluations are anticipated to be completed over a 24-month period (ie, by May 2028). Funding was received in August 2025; ethical approval was obtained on February 17, 2025; Clinical Trial Registry – India approval was obtained on March 18, 2025; and administrative approvals from the state government are in process. Preparatory phase activities (ie, obtaining ethical approval, Standard Operating Procedure development, project team recruitment, field site identification, and meeting with district stakeholders and the state government) were completed by March 2026. As there was a delay in initiation of the RCT component of the study, phases 1 and 2 have been initiated after obtaining required approvals at this time.

**Conclusions:**

This study will provide critical evidence on the burden of clinical/subclinical infections and AMR among children aged 6-59 months with uncomplicated SAM and inform the rational use of empirical antibiotics for CSAM amid growing AMR concerns.

**Trial Registration:**

Clinical Trials Registry India CTRI/2025/03/082358; https://ctri.nic.in/Clinicaltrials/pmaindet2.php?EncHid=MTIyMDI2&Enc=&userName=

## Introduction

### Background

Acute malnutrition is defined as a condition characterized by a severe nutrient deficit due to inadequate intake of food or the inability of the body to properly absorb essential nutrients [1]. Worldwide, 45.4 million children aged <5 years have wasting [2]. In India, the National Family Health Survey (NFHS)-5 reported a 19.3% burden of wasting and a 7.7% burden of severe wasting [3]. Although the burden of wasting remains almost constant, there is an increase in severe wasting compared to NFHS-4 and NFHS-3 [3-5]. Severe wasting indicates severe acute malnutrition (SAM) and is defined as a weight-for-height z-score (WHZ) of <−3 SD, a mid-upper arm circumference (MUAC) of <115 mm, or bilateral pitting pedal edema [6].

From the management perspective, SAM is further classified into complicated and uncomplicated SAM. Children with complicated SAM exhibit no appetite or have medical complications, while those with uncomplicated SAM have a successful standard appetite test and no fever, clinical infections, or complications [7]. Children with both complicated and uncomplicated SAM have a higher risk for acute infections, such as pneumonia, diarrhea, and sepsis, which triples the mortality risk: a few reported reasons are a weakened immune system, altered gut microbiota, poverty, food insecurity, and inadequate access to health care [8,9]. The fatality rate among children with SAM receiving inpatient care ranges from 3.4% to 35%. These data show that 3.5 million deaths are estimated among children in India aged <5 years [10].

In 2013, the World Health Organization (WHO) recommended inpatient management at nutritional rehabilitation centers using medications in line with the complications exhibited for children with complicated SAM and ambulatory Community-based Management of Severe Acute Malnutrition (CSAM) using energy-dense food and empirical antibiotics for children with uncomplicated SAM [11]. The empirical administration of broad-spectrum antibiotics, such as amoxicillin, as part of Community-based Management of Acute Malnutrition (CMAM) for children with uncomplicated SAM was a strong recommendation but with low certainty of evidence as per the 2013 guidelines [11]. However, there is a lack of high-quality evidence on the efficacy of empirical antibiotic therapy for 5-7 days among children with uncomplicated SAM at the community level. In settings with low infection rates among children with uncomplicated SAM, empirical management might lead to antibiotic overuse. Even in children aged <5 years with infection, empirical antibiotics might be ineffective due to poor compliance with medications and alarming levels of antibiotic resistance [12,13]. Furthermore, the overuse of empirical antibiotics among children with uncomplicated SAM can contribute to emerging antimicrobial resistance (AMR) in the community, especially among children [13]. AMR impairs the child’s development and second-line antimicrobials can contribute to lifelong disability, and worldwide estimates for 2019 indicate that 254,000 deaths of children are attributable to AMR [14]. The irrational prescribing of antibiotics and the suboptimal use of antimicrobials are the major contributors for AMR emergence [15-17]. Thus, further evidence on the relevance and appropriateness of empirical antibiotics for CSAM among children with uncomplicated SAM is required in the wake of increasing AMR.

### Rationale of the Study

In 2023, the Government of India released the National Protocol for Management of Malnutrition in Children to strengthen the ongoing efforts and commitment to address the challenges of malnutrition [18]. Mission Saksham Anganwadi and Poshan 2.0 was launched to drive the efforts to reduce malnutrition by identifying and managing children aged <5 years with SAM in India [19]. Under this program, peripheral health workers and *Anganwadi* (rural child care center in India) teachers are trained to make initial decisions on the level of inpatient or community-based care, nutrition management, and follow-up care for the identified children aged <5 years with SAM. These children are enrolled for CSAM at *Anganwadi*s and administered empirical amoxicillin for 5 days, twice a day, along with nutritional support for 4 months [20].

Although empirical antibiotics are currently in use, recent studies from the Africa region and one from India have not found them effective in improving nutritional recovery [21]. The potential reason was either a low rate of infection among children with uncomplicated SAM at the time of identification or AMR emergence, making empirical antibiotics ineffective [22-24]. Thus, the context-specific relevance of empirical antibiotics needed to be tested by assessing infection rates among children with uncomplicated SAM at the time of identification and by exploring antibiograms.

In India, a few observational studies in tertiary care hospitals among children with complicated SAM have reported a high prevalence of diarrhea (36%), fever (34%), and pneumonia (43%) [25-27]. However, this evidence does not warrant the use of empirical antibiotics among children with uncomplicated SAM managed at the community level without any clinical symptoms of infection. In addition, there is a lack of evidence on the burden of clinical/subclinical infection among children with uncomplicated SAM in India, and it is important to decide whether empirical antibiotics are necessary for this patient group.

A study [28] from India among children with sepsis in tertiary care centers reported that 48.2% and 90.3% of the children developed AMR to gentamicin and penicillin G, respectively. Another study [29] among pediatric patients in urban Delhi found high rates of AMR to commonly used antibiotics, such as amoxicillin and cotrimoxazole. Although the studies have reported high AMR prevalence among children, the literature on the burden of AMR among children aged <5 years with uncomplicated SAM in India is limited. Antibiograms and AMR profiles are necessary to decide whether the current choice of amoxicillin for empirical management of children with uncomplicated SAM is relevant.

Empirical antibiotics administered for 7 days can lead to poor medication compliance and suboptimal antibiotic use. In Niger, West Africa, empirical mass administration of a single dose of azithromycin was found feasible in a programmatic setting and was effective in reducing mortality due to meningitis, dysentery, malaria, and pneumonia among children aged <5 years [30,31]. A pilot randomized controlled trial (RCT) in Burkina Faso reported similar weight gain and fewer adverse events in the single-dose azithromycin group compared with the 7-day amoxicillin group [32]. However, azithromycin has not been tested in trial mode among children with uncomplicated SAM, and an RCT among children with uncomplicated SAM would generate evidence on the effectiveness of empirical antibiotics in nutritional recovery and aid in deciding whether a single dose of azithromycin is enough.

### Need for a Control Group With No Antibiotics

WHO recommends one course of amoxicillin for children with uncomplicated SAM; however, this is a conditional recommendation in both the 2013 and 2023 WHO guidelines on CSAM because of the low certainty of evidence [11,33]. The 2013 guideline was largely based on a single RCT [21] conducted in Malawi (2009-2011), which reported lower mortality in the amoxicillin group (4.63%) compared with the placebo group (7.48%). The trial included 924 children receiving amoxicillin, 923 receiving cefdinir, and 920 receiving a placebo. However, around 30% of the children had HIV, with no separate outcome analysis for HIV and non-HIV groups. Nearly 70% had kwashiorkor with edema, while only 20% had marasmus (WHZ<−3 SD). The study [21] was conducted in a high-infection setting, with 82% of children reporting fever, cough, or diarrhea prior to baseline, limiting the generalizability of its findings to settings with a lower infection burden.

However, a few other studies have reported different results. A double-blinded RCT [22] conducted by the Médecins Sans Frontières (MSF) in Niger (2012-2013) among children with uncomplicated SAM (n=1199 in the amoxicillin group and n=1200 in the placebo group) reported no difference in nutritional recovery between groups. Amoxicillin reduced early inpatient admissions within 2 weeks (14%), mainly by lowering diarrhea risk, slightly shortening the recovery time (28 vs 30 days, *P*<.001), and reducing mortality among children aged >2 years. However, by 8 weeks, there were no differences in death, default, or nonresponse between groups. Amoxicillin showed no benefit among children with confirmed bacterial infections, fever, or respiratory illness, and no differences were observed in inpatient admissions or recovery among hospitalized children. Hence, the authors concluded that amoxicillin does not play a significant role in nutritional recovery and that, as the study was conducted in a low-infection setting (10% bacterial gastroenteritis, 4% bacteremia, and 3% bacteriuria confirmed by blood, stool, and urine cultures), antibiotics can be recommended in context-specific settings with high rates of infection.

An RCT [34] conducted in Kanpur, India, among 100 children with uncomplicated SAM (amoxicillin vs placebo) reported no significant differences in clinical or anthropometric parameters between groups. A systematic review and meta-analysis [24] similarly reported that the evidence on empirical antibiotic usage among children with uncomplicated SAM is weak and may require urgent testing.

The Indian Council of Medical Research’s (ICMR) 2019 “Treatment Guidelines for Antimicrobial Use in Common Syndromes” recommend empirical antibiotic use in common illnesses, such as severe sepsis and septic shock, acute bacterial meningitis, community-acquired pneumonia, and necrotizing fasciitis, only when proven to be beneficial. The guidelines also suggest de-escalating or modifying therapy based on culture and drug susceptibility [35]. There is evidence of acute respiratory infection (ARI; 50%) [36] and diarrhea from the NFHS-5 (7.3%), ARI from the NFHS-5 (2.8%), and fever or ARI from the NFHS-5 (69%) [3]. However, there is no evidence in community-based studies of confirmed bacterial infection among children, and most infections among children aged <5 years are suspected to be of viral origin. A 2004 Lancet review [37] reported that the coverage offered by the aminopenicillins group of antibiotics is only 37% in the case of pediatric sepsis in Southeast Asia and the Pacific region [37].

Considering all this evidence, the rationale behind using empirical antibiotics requires urgent assessment, and there is a dire need for a control arm without antibiotics to assess the efficacy of empirical antibiotics in children with uncomplicated SAM. Therefore, this study aims to (1) estimate the clinical/subclinical infection and AMR patterns among children with uncomplicated SAM; (2) explore health-seeking behavior, antibiotic use, and care preferences among caregivers of pediatric patients and identify challenges and facilitators faced by health care providers in patient management; and (3) compare the effectiveness of single-dose azithromycin, 5-day amoxicillin, and no antibiotic therapy on nutritional recovery and clinical outcomes.

### Study Objectives

The primary objectives of this study are as follows:

- To describe the prevalence of clinical/subclinical infection and AMR patterns and determine their association with sociodemographic, clinical, and nutritional factors among children aged 6-59 months with uncomplicated SAM
- To explore health-seeking behavior, antibiotic usage patterns, and care preferences among caregivers of children aged 6-59 months with uncomplicated SAM and the challenges and facilitators for health care providers in managing these children
- To assess the efficacy of 7-day amoxicillin versus single-dose azithromycin versus no antibiotic, along with standard nutritional care, in weight gain and nutritional recovery among children aged 6-59 months with uncomplicated SAM

The secondary objective of this study is to compare medication safety (clinical adverse events) and adherence in two intervention arms (amoxicillin and azithromycin).

## Methods

### Patient and Public Involvement

Patients and the public were not involved in the trial design. However, children aged <5 years with uncomplicated SAM and their caregivers will participate in the study. Study findings will be shared with caregivers, community stakeholders, and program implementers through dissemination meetings and policy briefs.

### Study Design

This study protocol has been registered with Clinical Trial Registry – India (CTRI/2025/03/082358). The study will be conducted in three phases:

- Phase 1: cross-sectional, analytical community-based study with a laboratory component
- Phase 2: qualitative study using focus group discussions (FGDs) among caregivers and in-depth interviews (IDIs) of health care providers of children aged <5 years with uncomplicated SAM
- Phase 3: an open-label, parallel-group RCT

### Study Area

Phase 1 of the study will be conducted in Yadadri Bhuvangiri District, Telangana, India [38]. The district has a population of ~800,000, with 16% urban, 80% rural, and some tribal populations, with ~300 villages and 1 municipal corporation area. According to the 2011 Census, this district had 50,000 tribal people. Every month, ~50 new children with uncomplicated SAM and ~100 children with moderate acute malnutrition (MAM) are identified by *Anganwadi* teachers during routine monthly growth-monitoring drives, and the data are entered on the website of the Nutrition and Health Tracking System (NHTS) of the state government under the Supervised Supplementary Feeding Programme (SSFP) [20]. The identified children are provided with locally available nutrient- and energy-dense food, Balamruthuam Plus, an enhanced ready-to-use therapeutic food. Children with MAM receive this food for 2 months, while children with uncomplicated SAM receives it for 4 months. All children with uncomplicated SAM are administered a weight-adjusted dose of amoxicillin twice daily for 5 days.

Phase 2 of the study will be conducted both in the community and the hospital. In the community, caregivers of children with uncomplicated SAM will be contacted for participation in the FGDs. Caregivers who participate in the quantitative study on clinical/subclinical infections and AMR will be eligible for this phase. In the hospital setting—which includes primary health centers (PHCs), subcenters, and district and tertiary care centers—various health care providers, such as medical officers (MOs), auxiliary nurse midwives (ANMs), and pediatricians, will be contacted to participate in the study. IDIs will be conducted with them.

Phase 3 of the study will be carried out in the community via house-to-house visits, initially in Yadadri Bhuvangiri District and later in further districts as the sample gets saturated.

### Sample Size

#### Phase 1

Bhutta et al [8] reported that the rates of clinical infections among children with complicated SAM vary from 15% to 68%. Considering 50% prevalence of lower respiratory tract infection among children with uncomplicated SAM, 95% CIs, 10% relative precision, 1.5% design effect, 10% nonresponse rate, and 5% type I error, we will require 606 participants aged 6-59 months with uncomplicated SAM.

#### Phase 2

A purposive sampling strategy will be followed to select participants. Primary caregivers of children with uncomplicated SAM will be identified based on their volunteerism and participation. Yadadri Bhuvanagiri District comprises four project areas (Bhongiri, Aler, Mothkur, and Ramanapeta), each consisting of multiple sectors and each sector covering 25 *Anganwadi*s [38]. FGDs are planned at the project level to ensure adequate representation across geographic areas and variations in the distribution of children with uncomplicated SAM. Initially, two FGDs are planned per project area; however, considering the larger geographic coverage and higher caseload in two project areas, an additional FGDs will be conducted in each of these areas based on data flow. In total, 10-12 FGDs will be conducted to capture diversity in experiences and practices across the district. Each FGD will include 8-10 participants.

As the aim is also to explore barriers and facilitators related to the management of children with uncomplicated SAM, IDIs are planned to include service providers across different levels of the health system. This approach reflects the continuum of care, as a child with uncomplicated SAM encounters multiple providers at different points within the health system. Therefore, two IDIs will be conducted in each health care worker group: Accredited Social Health Activist (ASHA), *Anganwadi* teacher (center), ANM (subcenter), MO (PHC), and pediatrician (district hospital and tertiary care center).

The data saturation principle will guide the final number of FGDs and IDIs to be conducted. Saturation will be considered achieved when no new themes, patterns, or insights emerge from successive FGDs or IDIs and when there is redundancy in the information obtained. Data collection and analysis will be undertaken simultaneously to monitor saturation, and additional FGDs or IDIs will be conducted, if required.

#### Phase 3

Rao et al [34] reported the WHZ mean (SD) as –1.29 (0.84) in the amoxicillin group and –1.45 (0.93) in the control group at the end of 2 weeks of intervention. Considering these values, with 95% CI and 80% power, we will require 480 participants in each group. Considering logistic issues, feasibility, and long follow-up, we will recruit 250 children aged 6-59 months with uncomplicated SAM per group.

### Inclusion and Exclusion Criteria

#### Phase 1

The inclusion criteria are (1) age 6-59 months, (2) both sexes, (3) resident of the study area, (4) WHZ≤–3 SD without any signs of complications (uncomplicated SAM), and (5) passing the appetite test.

Children with SAM who have medical complications or infection have loss of appetite due to underlying physiological changes. The appetite test helps identify such children. Every child identified as having SAM during growth monitoring will undergo the appetite test to decide the further course of treatment. In Telangana, Balamrutham Plus is used under the SSFP for the test. The quantity provided varies by age: age 7-18 months, at least 15 g; age 19-36 months, 30 g; and age 37-59 months, 50 g. Children who are able to consume the given quantity are considered to have passed the test and are enrolled in the SSFP; otherwise, they are referred to a nutrition rehabilitation center for further treatment. The test usually takes a short time but may even take up to 1 hour [20].

The exclusion criteria are (1) WHZ≥–2 SD, between –2 SD and –3 SD, or <–3 SD with complications (children with complicated SAM); (2) bilateral pitting edema; (3) congenital anomalies, inborn errors of metabolism, or developmental delay; and (4) malabsorption syndromes.

Children with SAM will be identified from the NHTS through the district health administration. During the study period, all eligible children will be contacted to participate in the study.

#### Phase 2

Primary caregivers of children aged 6-59 months with uncomplicated SAM will be identified based on their volunteerism and participation. Each FGD will include 8-10 participants, and 10-12 FGDs will be conducted, in addition to 2 IDIs in each health care worker group: ASHA, *Anganwadi* teacher (center), ANM (subcenter), MO (PHC), and pediatrician (district hospital and tertiary care center). The number of FGDs and IDIs will be based on the level of information saturation.

#### Phase 3

The inclusion criteria are (1) age 6-59 months, (2) enrollment in the SSFP/CMAM program, (3) WHZ <–3 SD or MUAC=115 mm, (4) passing the appetite test using Balamrutham Plus, (5) both sexes, (6) available for the entire 6 months of the study period, (7) no edema, (8) no antibiotic use in the past 1 month, (9) no clinical complications requiring antibiotic treatment and/or inpatient care, and (10) primary resident of the catchment area. Children who participate in phase 1 will also be eligible to be part of phase 3.

The exclusion criteria are (1) complicated cases of SAM and (2) antibiotic use in the past 1 month.

### Study Procedure

**Figure 1:**
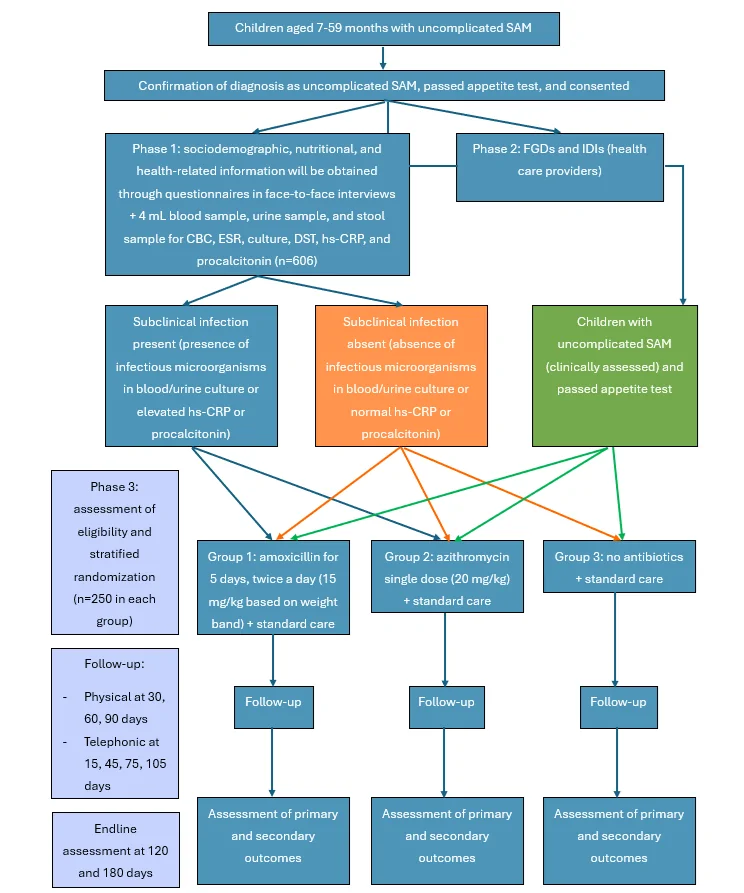
RCT CONSORT flow diagram for recruitment, follow-up, and endline assessment of participants in the study. CBC: complete blood count; CONSORT: Consolidated Standards of Reporting Trials; DST: drug susceptibility testing; ESR: erythrocyte sedimentation rate; FGD: focus group discussion; hs-CRP: high-sensitivity C-reactive protein; IDI: in-depth interview; RCT: randomized controlled trial; SAM: severe acute malnutrition.

Figure 1 shows the CONSORT (Consolidated Standards of Reporting Trials)–based RCT flow diagram depicting the enrollment of participants for each phase of the study: randomization, follow-up, and endline assessment. Table 1 depicts the timeline of the trial activities.

**Table 1:** Timepoints and activities to be carried out during the study.

Activity	Baseline	Enrollment to RCT^a^ (within 7 days of collecting baseline data)	Telephonic follow-up at 15, 45, 75, and 105 days (plus 5 days allowed at each timepoint)	Physical follow-up at 30, 60, 90, 120, and 180 days (plus 5 days allowed at each timepoint)
Consent; appetite test; sociodemographic, nutritional, and health-related information obtained through questionnaires during face-to-face interviews + 4 mL^b^ of blood sample, urine sample, and stool sample for CBC^c^, ESR^d^, culture, DST^e^, hs-CRP^f^, procalcitonin, and anthropometry (height, weight, MUAC^g^)	✓	—^h^	—	—
RCT eligibility assessment, randomization and antibiotic distribution, and IFA^i^ syrup distribution in the case of anemia	—	✓	—	—
Weight, height, MUAC, symptoms of adverse events, any illness, and treatment details	—	—	—	✓
Symptoms of adverse events, any illness, and treatment details	—	—	✓	✓

^a^RCT: randomized controlled trial.

^b^Biological samples will be collected from 606 study participants.

^c^CBC: complete blood count.

^d^ESR: erythrocyte sedimentation rate.

^e^DST: drug susceptibility testing.

^f^hs-CRP: high-sensitivity C-reactive protein.

^g^MUAC: mid-upper arm circumference.

^h^Not applicable.

^i^IFA: iron folic acid.

#### Phase 1

A list of children with uncomplicated SAM will be obtained from the NHTS website through district stakeholders. The children will be visited at their homes to confirm their diagnosis and malnutrition category and will be enrolled into the study after obtaining informed consent from the caregivers. The children’s sociodemographic, nutritional, and health-related information will be obtained through questionnaires and interviews. Under a sterile environment, 4 mL of blood sample will be taken from each child. Two sterile containers will be given to the children to collect urine and stool samples, and those samples will be collected from the caregivers the following day.

Blood samples will be transported in 2-8^o^C and will be sent to the biochemistry laboratory within 4 hours of sample collection. The samples will be analyzed in the biochemistry (high-sensitivity C-reactive protein [hs-CRP], procalcitonin) and pathology (complete blood count [CBC], erythrocyte sedimentation rate [ESR], and white blood cell [WBC] count) laboratories. Blood, stool, and urine samples will be analyzed in the microbiology laboratory for culture and sensitivity, and urine samples will additionally be analyzed for complete urine examination in the pathology laboratory. To capture the mental health status of both parents, consent from both will be obtained.

#### Phase 2

FGD and IDI guides (Multimedia Appendices 1 and 2) will be prepared based on the literature review and the knowledge gap requiring further data [39]. Potential participants for qualitative interviews will be identified and appointments will be made. A moderator trained in qualitative research will conduct the FDGs and IDIs. The date and time of the FGDs and IDIs will be fixed at the participants’ convenience. On the day of the FGDs and IDIs, informed consent will be obtained, and the entire process will be audio-recorded. An assistant will take field notes, and a sociogram will be used to map the participation pattern. Participatory rural appraisal techniques will be used to encourage active participation and generate more discussion among the participants in the FGDs. The duration of each FGD and IDI will be 30-45 minutes.

#### Phase 3

A list of children with uncomplicated SAM will be obtained from the NHTS website. The children will be visited at their homes to confirm their nutritional category. At enrollment, each child will be assigned a unique identification number based on the number in the list. After baseline assessment, children will be randomized using stratified randomization to one of three groups: amoxicillin (5 days, twice a day, 15 mg/kg based on weight band + standard nutritional care), azithromycin (single dose, 20 mg/kg + standard nutritional care), or control (no antibiotics + standard nutritional care). Children with subclinical infection will be randomized to either the amoxicillin or the azithromycin group in a 1:1 allocation ratio. Children without any subclinical infection will be randomized to the amoxicillin, azithromycin, or control group in a 1:1:2 randomization sequence to balance the stratified randomization based on subclinical infection status. The remaining children who will be assessed clinically for uncomplicated SAM, who pass the appetite test, and who are found eligible will be assigned to one of the three groups in a 1:1:1 randomization sequence; these children will be recruited after completion of the phase 1 enrollment. Around 150-200 participants are expected to be recruited after phase 1 enrollment to fulfill the sample size requirement for phase 3.

Three separate randomization sequences of unequal block sizes will be generated. A non–peer-reviewed study [40] from Bangladesh reported that 36% of children with uncomplicated SAM have elevated CRP and α1-glycoprotein. Assuming that 35% of children with uncomplicated SAM might have a subclinical infection [40], a randomization sequence will be generated using online software. The allocation will be concealed centrally in the institute. Based on the intervention plan, study participants and administrators will not be masked in the study.

### Preparatory Phase

In this phase, the investigator and co-investigators will form a technical advisory group, following which subject matter experts will be involved in RCT-related activities (including the final protocol and Standard Operating Procedures [SOPs] for implementing the intervention). The implementation plan will involve identifying blocks, the data collection process, quality monitoring, the safety reporting mechanism, management of adverse events, and ethical considerations. This period will also be used to recruit the project team and closely work with district stakeholders and the state government for approval of implementation.

### Intervention Phase

Following eligibility testing, children will be enrolled in one of the three groups based on stratified randomization. Baseline data of various independent and dependent variables will be captured for each child with proper screening of anthropometric measures and medical assessment. Next, each child will receive either oral amoxicillin or azithromycin and standard nutritional care (Balamrutham Plus) based on the group. In addition, based on the hemoglobin level, children with anemia at baseline will be administered iron folic acid (IFA) syrup as per Anemia Mukt Bharat guidelines [41]. Any illness during the intervention period, physician (or pediatrician) visits, treatment details, and hospitalization details will be documented during the follow-up visits.

In the amoxicillin group, amoxicillin dispersible tablet (125 mg) will be administered, along with Balamrutham Plus. The first dose will be administered upon enrollment, following by medication at home (for 5 days, twice daily). The dosage will be determined based on weight of the child, as specified by the SSFP implemented by the Government of Telangana for managing children with SAM in all districts of Telangana (Table 2) [20].

**Table 2:** Dosage of amoxicillin dispersible tablets.

Weight of child (kg)	Prescription	Tablets required, n
4.0-6.9	1 tablet twice daily for 5 days	10
7.0-9.9	1.5 tablets twice daily for 5 days	15
10.0-12.9	2 tablets twice daily for 5 days	20
13.0-15.9	2.5 tablets twice daily for 5 days	25
16.0-18.9	2.5 tablets twice daily for 5 days	25

In the azithromycin group, a single dose of azithromycin syrup (20 mg/kg) will be administered, along with Balamrutham Plus.

In the control group, no antibiotics will be prescribed; only standard nutritional treatment (Balamrutham Plus) will be provided.

#### Follow-Up Visits

Children will be followed up monthly during the intervention, in addition to follow-up postdischarge from the program at 4 and 6 months. Telephone calls will be made once every 15 days to document any illness, medication use, or side effects. Physical visits will be made every 30, 60, 90, 120 and 180 days.

For compliance assessment, empty amoxicillin strips will be collected at 30 days. For azithromycin, single-dose compliance assessment will be performed on the day of enrollment to the RCT. An additional buffer of 5 days will be allowed to collect data at each timepoint. Clinical adverse events of the intervention will be assessed during physical visits and documented over the phone.

### Operational Definitions

Children with uncomplicated SAM are clinically well (ie, without signs of infection) and with a retained appetite (passed the appetite test), which is considered indicative of the absence of severe metabolic disturbance. SAM is defined as WHZ<–3 SD of the median WHO child growth standards and/or by the presence of bilateral pitting edema. Nonresponse to treatment is defined as children who do not meet discharge criteria (WHZ>–2 SD) after 4 months.

### Outcomes

The primary outcomes of the RCT are as follows:

- Weight gain at 16 weeks and 6 months after intervention initiation
- WHZ at 16 weeks and 6 months
- MUAC at 16 weeks and 6 months
- Anthropometric measurements collected by trained study staff using standardized procedures and calibrated equipment: weight measured using digital weighing scales; height/length using stadiometers or infantometers, as appropriate; and MUAC using standardized MUAC tapes

The secondary outcomes include the following:

- Time to nutritional recovery
- Nutritional status at follow-up, categorized as cured (WHZ>−2 SD), nonresponder (WHZ<−2 SD), or lost to follow-up
- Transfer to inpatient care
- Clinical signs of infection and morbidity episodes, including fever, diarrhea, and respiratory symptoms
- Recurrent infections during follow-up
- Hemoglobin levels
- Adverse events related to antibiotic therapy
- Mortality

Clinical outcomes and morbidity episodes will be assessed during scheduled follow-up visits through caregiver interviews and clinical examination by trained study personnel. Hemoglobin levels will be estimated using standard laboratory methods. Information related to inpatient referrals, adverse events, and mortality will be documented using study follow-up records.

The exploratory outcomes include the following:

- Proportion of children with clinical infections based on clinical evaluation results
- Proportion of children with subclinical infections assessed using hs-CRP, procalcitonin levels, blood culture, and urine culture
- Growth in stool culture
- Patterns of antimicrobial sensitivity and resistance among children with uncomplicated SAM
- Health-seeking behavior, antibiotic usage patterns, and care preferences among caregivers of children with uncomplicated SAM
- Challenges and facilitators in the management of uncomplicated SAM, as perceived by health care providers

Blood, urine, and stool samples will be collected and processed using standard microbiological and laboratory procedures to assess infection status and AMR patterns. Qualitative data will be collected through FGDs and IDIs using semistructured interview guides.

### Covariates and Other Assessments

The following covariates will be assessed:

- Day care absenteeism
- Parental absenteeism
- Mental health status of parents
- Socioeconomic status
- Domestic violence
- Food consumption patterns
- Household food insecurity

Information about day care absenteeism will be obtained from Integrated Child Development Services records. Socioeconomic status will be assessed using the BG Prasad socioeconomic scale. Dietary intake will be assessed using a 24-hour dietary recall method. Household food insecurity will be assessed using the Household Food Insecurity Access Scale and categorized into food-secure, mildly food-insecure, moderately food-insecure, and severely food-insecure households. Questions adapted from the NFHS-5 women’s questionnaire will be used to assess domestic violence.

### Data Entry and Analysis

#### Phase 1

Data will be entered into the Epicollect5 mobile app and analyzed in Stata version 18.0 software. Continuous variables (age, weight, height, WBC count, ESR, hs-CRP, procalcitonin, and colony-forming units) will be expressed as mean (SD) or median (IQR) based on data distribution. Categorical variables (clinical infection, subclinical infection, and AMR) will be expressed as percentages. The association between independent factors (sociodemographic, health, and nutritional factors) and of clinical/subclinical infection and AMR outcomes will be analyzed using chi-square tests, *t* tests, or Mann-Whitney *U* tests. Multivariate analysis using log-binomial regression will be performed to adjust for confounders. *P*<.05 will be considered statistically significant.

#### Phase 2

English transcripts will be prepared within 24 hours of conducting an IDI/FGD. Qualitative data will be analyzed in Atlas Ti software. First, complete transcripts will be read by the investigators to ensure immersion, initial codes will be generated inductively using inductive coding in Atlas.ti software, and the codes will be iteratively refined through discussions and organized into subcategories (subthemes) and higher-order categories (themes) in line with reflexive thematic analysis principles [42]. An audit trail (coding decisions, evolving codebooks, and notes from analytical discussions) will be maintained throughout the process to support confirmability and dependability. We will also perform data triangulation of qualitative and quantitative data to increase the credibility of the information collected about knowledge, barriers, and facilitators related to antibiotic usage among children with uncomplicated SAM by adopting convergent parallel mixed methods design.

#### Phase 3

Data analysis will be performed in Stata version 18.0 software using intention-to-treat analysis at the end of the study. Per protocol analysis will also be performed as a secondary analysis at the end of the study. Interim analysis will be part of the quality parameter (at the end of the first year). The WHZ and MUAC will be expressed as mean (SD) or median (IQR) with 95% CIs based on data normality. The presence of bilateral pitting edema will be expressed as proportions with 95% CIs. Independent variables (child’s age in months and mother’s age) will be expressed as mean (SD) or median (IQR) with 95% CIs based on data normality. Factors such as education status, type of delivery, and birth complications will be expressed as proportions with 95% CIs. Outcomes such as episodes of acute illness, day care absenteeism, parenteral absenteeism, number of hospital visits, and food consumption will be expressed as mean (SD) or median (IQR) with 95% CIs based on data normality. Outcomes such as the presence of sepsis and mortality status will be expressed as proportions with 95% CIs. The chi-square test will be performed to analyze the association between outcomes and categorical independent variables. An unpaired *t* test (or Mann-Whitney *U* test) will be performed to assess the association between outcomes and continuous independent variables. Poisson regression analysis (bivariate and multivariate) will be conducted to explore the association between dependent and independent factors. Adjusted risk ratios with 95% CIs will be reported for factors included in the multivariate Poisson regression model.

### Ethical Considerations

This study protocol has been reviewed and approved by the Institutional Ethics Committee (IEC) and the Clinical Trial Review Committee (CTRC) of the All India Institute of Medical Sciences (AIIMS), Bibinagar, India (IEC approval #AIIMS/BBN/IEC/Feb/2025/611-R; CTRC approval #2024/12/08). Any important modifications to the study protocol, including changes to eligibility criteria, interventions, outcomes, or procedures, will be communicated promptly to the IEC, trial registries, and investigators. Participants and their guardians will be informed if the changes affect their participation.

Signed informed consent will be obtained from the parents or legal guardians of all participating children in the presence of a witness, along with the witness’s signature, prior to enrollment. Trained study staff will explain the study purpose, procedures, potential risks, and benefits in the local language and provide a patient information sheet in the local language. Parents/guardians will also be informed that participation is voluntary and that they have the right to withdraw from the study at any time without any consequences related to access to health care services.

Participant confidentiality will be strictly maintained. Personal identifiers will be stored separately from study data in secure, password-protected systems accessible only to authorized personnel. Any data shared for analysis or publication will be deidentified. Paper records, if any, will be kept in locked cabinets within the study site. An independent committee (Data & Safety Monitoring Board) will be established to ensure safety and review protocol amendments, data quality, completeness, compliance with the intervention, and other information relevant to the study.

During the study period, if any participants fall ill, they will be immediately referred to a nearby PHC, health center, or tertiary care center (AIIMS, Bibinagar) based on the care provider’s preference to provide care. Any medication use during the study period will be documented. No monetary compensation will be provided to participants for participation in the study.

## Results

The study was funded by the ICMR in August 2025. Ethical approval was obtained from the IEC of AIIMS, Bibinagar, on February 17, 2025, and CTRI approval was obtained on March 18, 2025; administrative approvals from the state government are in process.

Participant recruitment began in June 2026. Baseline assessments, interventions, and final evaluations are anticipated to be completed over a 24-month period (ie, by May 2028). As of March 2026, preparatory phase activities have been completed: obtaining ethical approvals, SOP development, project team recruitment, field site identification, and meeting with district stakeholders and the state government. As there is a delay in the initiation of the RCT component of the study, phases 1 and 2 have been initiated after obtaining required approvals at this time. The results of this study will be disseminated through peer-reviewed publications, stakeholder consultations, and policy briefs, with the aim of informing national guidelines and strengthening evidence-based management of children with uncomplicated SAM in community settings.

## Discussion

### Summary

This RCT is designed to estimate the prevalence of clinical and subclinical infections and AMR patterns among children with uncomplicated SAM in community-based settings. Further, the study aims to evaluate the necessity and effectiveness of empirical antibiotic therapy in this population.

The current recommendation outlined by WHO for routine empirical antibiotic use in children with uncomplicated SAM is conditional, based on low-certainty evidence, primarily derived from high-infection settings. Subsequent studies from sub-Saharan Africa and limited evidence from India have demonstrated inconsistent findings regarding the benefits of antibiotics in improving nutritional recovery. Notably, trials conducted in relatively low-infection settings have demonstrated no significant differences in recovery outcomes between antibiotic and placebo groups.

### Strengths and Limitations

In the Indian context, evidence is scarce for community-based management settings in which children with uncomplicated SAM are less likely to present with overt infections. Simultaneously, increasing AMR to commonly used antibiotics raises concerns about the continued empirical use of such drugs without clear benefits. This study is, therefore, expected to generate context-specific evidence to inform more rational and targeted use of antibiotics in the CSAM in the Indian context. The RCT design with three arms—amoxicillin, azithromycin, and no antibiotics—will allow for a comprehensive evaluation of both the effectiveness and the necessity of empirical antibiotic therapy, which is one of the major strengths of this study. Moreover, the study is embedded within a community-based management framework, enhancing its relevance to current national programs and real-world implementation. In addition, the inclusion of laboratory investigations to assess infection status and AMR provides a stronger evidence base that is often lacking in similar studies.

However, the study will be conducted within specific programmatic and geographic settings, which may limit generalizability to regions with differing infection burdens or health care access. Moreover, subclinical infections may not be fully captured due to inherent limitations in diagnostic methods. Operational challenges, such as timely collection and transportation of samples, particularly urine and stool samples among pediatric participants, may be difficult. In addition, adherence to treatment and follow-up in community settings may also influence study outcomes.

The findings of this study have important implications for both policy and practice. If empirical antibiotics are found to provide limited benefits in children with uncomplicated SAM, this could support revisiting existing guidelines and promoting more judicious use of antibiotics, in line with antimicrobial stewardship principles. However, if benefits are observed in specific subgroups, the study may help refine criteria for targeted antibiotic use.

### Conclusion

The evaluation of single-dose azithromycin as an alternative therapy regimen for SAM may offer operational advantages, particularly in improving medication adherence and reducing the treatment burden in large-scale programs implemented through community platforms, such as *Anganwadi*s in India. Future research should focus on longitudinal studies to further explore the impact of antibiotic use on relapse rates, growth outcomes, and AMR patterns over time.

## Data Availability

The data sets of this study will not be available for open access publicly and access will be restricted as the data contain sensitive health and other information of study participants. Data will be available with the corresponding author and would be shared in deidentified format upon reasonable request and clearance from the Institutional Ethics Committee, the Clinical Trial Review Committee, and relevant authorities.

## Appendix

Multimedia Appendix 1 FGD guide to explore health-seeking behavior, antibiotic usage patterns, and care preferences among caregivers of children aged 6-59 months with uncomplicated SAM. FGD: focus group discussion; SAM: severe acute malnutrition.

Multimedia Appendix 2 IDI guide to explore health care providers’ challenges and facilitators in managing children with uncomplicated SAM under the CMAM program in Telangana, India. CMAM: community-based management of moderate acute malnutrition; IDI: in-depth interview; SAM: severe acute malnutrition.

Multimedia Appendix 3 Peer review report by the Indian Council of Medical Research (ICMR).

## Abbreviations

AMR: antimicrobial resistance
ANM: auxiliary nurse midwife
ARI: acute respiratory infection
ASHA: Accredited Social Health Activist
CBC: complete blood count
CMAM: Community-based Management of Acute Malnutrition
CONSORT: Consolidated Standards of Reporting Trials
CSAM: Community-based Management of Severe Acute Malnutrition
CTRC: Clinical Trial Review Committee
CTRI: Clinical Trial Registry – India
DST: drug susceptibility testing
ESR: erythrocyte sedimentation rate
FGD: focus group discussion
hs-CRP: high-sensitivity C-reactive protein
ICMR: Indian Council of Medical Research
IDI: in-depth interview
IEC: Institutional Ethics Committee
IFA: iron folic acid
MAM: moderate acute malnutrition
MO: medical officer
MUAC: mid-upper arm circumference
NFHS: National family health survey
NHTS: Nutrition and Health Tracking System
PHC: primary health center
RCT: randomized controlled trial
SAM: severe acute malnutrition
SOP: Standard Operating Procedure
SSFP: Supervised Supplementary Feeding Programme
hs-CRP: high-sensitivity C-reactive protein
WBC: white blood cell
WHO: World Health Organization
WHZ: weight-for-height z-score
