# Supplementary material for: Efficacy of Empirical Antibiotics in Community-Based Management of Uncomplicated Severe Acute Malnutrition in Children: Protocol of a Randomized Controlled Trial
Source: JMIR Res Protoc. 2026 Sep 2;15:e91579. doi: 10.2196/91579 (PMC13583509; doi:10.2196/91579)
Supplement: Supplementary file 1 [file resprot_v15i1e91579_app3.pdf]

Gmail

Gomathi Ramaswamy <gmthramaswamy@gmail.com>

ICMR: Proposal Id IIRPSG-2024-01-05785 result (no-reply to this email)

no-reply@hq.icmr.org.in <no-reply@hq.icmr.org.in> Wed, Oct 30, 2024 at 3:26 PM
To: Dr Gomathi Ramaswamy <gmthramaswamy@gmail.com>
Cc: ICMR-ePMS <po.epms@icmr.gov.in>, ICMR-ePMS <aparna.sinha.deb@icmr.gov.in>, ICMR-ePMS <no-reply@hq.icmr.org.in>

Dear Dr Gomathi Ramaswamy,

This is with reference to your proposal Id “**IIRPSG-2024-01-05785**”, titled “**Is empirical antibiotics necessary for Community-based management of Severe Acute Malnutrition (CSAM) in children. Can single-dose Azithromycin replace one-week Amoxycillin for CSAM: Randomized-Control trial**”.

We are pleased to inform you that your project has been approved, subject to satisfactory response to the following technical and budgetary comments (as applicable).

Technical Comments: The title and objective include SAM, however, the sampling frame includes both SAM and MAM, which needs to be corrected. The ethical implications of withholding empirical antibiotics in the third arm has to be detailed and justified, within two weeks of receipt of this email, please upload a revised proposal addressing the technical comments. For ease of review do highlight the revisions.

Budgetary Comments: Nil, PI to prepare a justification of budget and response to budgetary comments for a VC meeting with Budget Review Committee to be held within the next two weeks of receipt of the email.

We would also ask you to urgently arrange for all the CODAL formalities to be completed as per the current Guidelines for Extramural Research Programme of ICMR. ***Please submit for IEC/ regulatory approvals (as applicable) immediately.***

If the documents pertaining to CODAL formalities are not uploaded in ICMR-ePMS portal within the stipulated period (not later than 3 months of this email),the approval may be cancelled.

For any clarification/s on the technical or budgetary comments, please contact "Dr. Aparna Mukherjee (aparna.sinha.deb@icmr.gov.in)"

Important Note: This is an automated email. Kindly refrain from replying to this email.

Warm Regards
Program Officer

e-Project Management System
Indian Council of Medical Research
Department of Health Research
Ministry of Health & Family Welfare
Government of India
Ansari Nagar, New Delhi - 110029, India
www.icmr.gov.in

</html
