# Supplementary material for: Efficacy of Empirical Antibiotics in Community-Based Management of Uncomplicated Severe Acute Malnutrition in Children: Protocol of a Randomized Controlled Trial
Source: JMIR Res Protoc. 2026 Sep 2;15:e91579. doi: 10.2196/91579 (PMC13583509; doi:10.2196/91579)
Supplement: Supplementary file 2 [file resprot_v15i1e91579_app1.docx]

**FOCUS GROUP DISCUSSION (FGD) GUIDE**

**Objectives:** To explore health-seeking behavior, antibiotic usage pattern and preference for care among the caregivers of children aged 6-59 months with uncomplicated SAM.

**Participants:** Caregivers of Children Aged 6–59 Months with Severe Acute Malnutrition (SAM)

**Ideal Group Size:** 8–10 participants.

**Materials Needed:** Chart paper, colored markers, seeds/stones (counters), local objects [syrup bottles, Ready to Use Therapeutic Feeding (RUTF) packets], and pictorial cards representing symptoms and providers.

**FGD Details:**

1. **Date:** ___________________________
2. **Starting Time:** ___________________
3. **Ending Time:** _____________________
4. **District:** _________________________
5. **Sector/Block:** _____________________
6. **Project Name:** ____________________
7. **Venue of FGD:** __________________________________________
8. **Name of Facilitator/Moderator:** __________________________
9. **Name of Note Taker:** ____________________________________
10. **Name of Observer (if any):** ______________________________

**Instructions for Moderator/Interviewer *(Do not read aloud)***

- Ensure availability of all materials prior to the discussion:
  consent forms, audio recorder, notebook, writing & Participatory Rural Appraisal (PRA) materials (pen/ink pad for thumb impressions).
- Questions and probes may be rephrased to maintain a natural flow of discussion.
- It is not mandatory to ask every probe; however, ensure all key themes are adequately covered.
- Encourage participation from all group members and maintain a neutral, non-judgmental approach.

**Step 1: Introduction & Briefing of the study**

- Greet participants warmly. Introduce yourself, the note-taker, and observer (if present). Establish rapport and create a comfortable discussion environment.
- Briefly explain the purpose, objectives of the study, also clarify why participants have been selected and inform them about the expected duration of the discussion.

**Step 2: Informed Consent**

- Read the informed consent form aloud in the local language. Clarify any questions or doubts raised by participants.
- Obtain written consent or thumb impression from each participant before proceeding.

**Step 3: Participant Characteristics**

| **Participant ID** | **P1** | **P2** | **P3** | **P4** | **P5** | **P6** | **P7** | **P8** | **P9** | **P10** |
| --- | --- | --- | --- | --- | --- | --- | --- | --- | --- | --- |
| Age |  |  |  |  |  |  |  |  |  |  |
| Sex |  |  |  |  |  |  |  |  |  |  |
| Marital Status |  |  |  |  |  |  |  |  |  |  |
| Education |  |  |  |  |  |  |  |  |  |  |
| Occupation |  |  |  |  |  |  |  |  |  |  |

**Coding for Participant Characteristics**

- **Sex:**
  1 = Male, 2 = Female
- **Marital Status:**
  1 = Single, 2 = Married, 3 = Divorced/Separated, 4 = Widowed
- **Education Level:**
  1 = No formal schooling, 2 = Primary education, 3 = Secondary education, 4 = Higher education
- **Occupation:**
  1 = Not working/Household work/Farmer, 2 = Business, 3 = Employed (formal/informal),

4 = Others (specify)

**Section 2: FGD Thematic Questions with PRA Techniques**

**Icebreaker & Rapport Building**

**Icebreaker & Rapport Building**

Technique: Seasonal Mapping (Health Calendar)

Objective: To understand seasonal patterns of child illnesses and initiate discussion comfortably.

Process:

- Ask participants to draw a “Health Calendar” using stones/seeds.
- Mark months when children fall sick more frequently.

Probes:

- In which months do children fall sick most often?
- What are the common illnesses during these periods?
- Are certain illnesses linked to seasons (rainy, summer, winter)?

**Section – 1 : Understanding and Local Terminology of Malnutrition**

Technique: Free Listing and Pile Sorting

Objective: To explore caregivers’ understanding and local terminology of malnutrition.

Process:

- Ask: “What do you understand by malnutrition?”
- Record responses on cards/slips.
- Ask: “What is the local name for malnutrition?”
- Conduct pile sorting into categories (types/severity).

Probes:

- Differences between weakness, thinness, and illness
- Local beliefs or misconceptions
- Severity classification (mild/moderate/severe)

**Section – 2: Identification of Signs and Symptoms**

Technique: Body Mapping / Visual Listing

Objective: To identify caregivers’ recognition of signs and symptoms of malnutrition.

Process:

- Draw a child’s outline.
- Ask participants to mark features of a malnourished child.

Probes:

- Thin arms/legs (wasting)
- Swelling (edema)
- Hair changes
- Reduced activity/playfulness
- Loss of appetite

**Section – 3: Causes of Malnutrition**

Technique: Problem Tree Analysis

Objective: To explore perceived causes of malnutrition.

Process:

- Write: “Child becomes malnourished”
- Ask participants to list causes (roots).

Probes:

- Feeding practices
- Illness (diarrhea, fever, infections)
- Poverty/food insecurity
- Lack of awareness
- Care practices

**Section – 4: Treatability and Care Practices**

Technique: Venn Diagram / Solution Mapping

Objective: To understand perceptions regarding treatment and care of malnutrition.

Process:

- Ask: “Can malnutrition be treated?”
- Map available treatment options and care practices.

Probes:

- Types of foods given
- Use of Take Home Ration (THR)/supplementary nutrition
- Medical treatment
- Beliefs about recovery

**Section – 5: Health-Seeking Behaviour Pathway**

Technique: Care Pathway Mapping (Flow Diagram)

Objective: To map the sequence of care-seeking actions during child illness.

Process:

- Ask: “When your child becomes sick, what do you do first?”
- Develop a flow diagram of care pathways.
- Highlight where medicines (especially antibiotics) are introduced.

Probes:

- First point of care
- Time taken to seek care
- Decision-makers in the household
- Role of Anganwadi Worker (AWW) /Accredited Social Health Activist **(**ASHA)
- Barriers (cost, distance, access)

**Section – 6: Preferences for Treatment and Care**

Technique: Preference Ranking / Scoring Matrix

Objective: To assess caregiver preferences for healthcare providers.

Process:

- List healthcare options.
- Ask participants to score based on cost, access, quality, and trust.

Probes:

- Government vs private preference
- Trust and satisfaction
- Role of frontline workers
- Expectations from services

**Section – 7: Barriers and Facilitators in Child Care**

Technique: Problem Tree

Objective: To identify barriers and facilitators affecting child recovery under Community based treatment of Severe acute malnutrition (CSAM)

Process:

- Core problem: “Child not improving”
- Identify roots (barriers) and branches (consequences).
- List facilitators.

Probes:

- Access issues
- Cost constraints
- Beliefs and practices
- Medicine adherence
- Support systems (AWW/ASHA)

**Section – 8: Antibiotic Use Practices**

Technique: Drug Identification and Use Mapping

Objective: To understand patterns of antibiotic use in children.

Process:

- Ask participants to list commonly used medicines.
- Categorize into antibiotics vs non-antibiotics.

Probes:

- Awareness of antibiotics
- Prescriber [doctor/pharmacist/self/informal health providers/ Rural Medical Practitioners (RMP)]
- Completion of course
- Use of leftover/shared medicines
- Situations of antibiotic use

**Section – 9: Perceptions of Antibiotics in Child Recovery**

Technique: Agree–Disagree Matrix

Objective: To explore beliefs and perceptions about antibiotics.

Process:

- Present statements and ask participants to agree/disagree.

Probes (Statements):

- All weak children need antibiotics
- Medicines can be stopped once the child improves
- One dose of medicine is enough
- Antibiotics help faster recovery

**Section – 10: Adherence and Feasibility of Antibiotic Regimens**

Technique: Treatment Preference Ranking

Objective: To assess feasibility and preference for different antibiotic regimens.

Process:

- Present options:
  1. 7-day course
  2. Single-dose
  3. No antibiotic
- Ask participants to rank options.

Probes:

- Ease of administration
- Trust
- Perceived effectiveness
- Challenges (taste, vomiting, forgetting doses)

**Section – 11: Acceptability of No Antibiotic Approach**

Technique: Scenario-Based Discussion

Objective: To assess acceptability of withholding antibiotics in uncomplicated SAM.

Process:

- Present scenario: “A child with malnutrition but no infection is advised only nutrition therapy.”

Probes:

- Would you accept this advice? Why/why not?
- Trust in doctor’s decision
- Fear of worsening illness
- Expectations for medicines

**Section – 12: Barriers to Appropriate Antibiotic Use**

Technique: Pairwise Ranking

Objective: To identify key barriers to appropriate antibiotic use.

Process:

- List barriers and ask participants to rank them.

Probes:

- Cost
- Access
- Lack of guidance
- Misuse due to low awareness

**Section – 13: Role of Health System and Frontline Workers**

Technique: Guided Discussion

Objective: To understand the role of health providers in influencing treatment practices.

Process:

- Ask participants about advice received from providers.

Probes:

- Counseling on medicines
- Trust in public health system
- Follow-up and support

**Section – 14: Suggestions for Improving SAM Treatment**

Technique: Solution Mapping

Objective: To gather participant-driven recommendations for improving treatment adherence and outcomes.

Process:

- Ask participants to suggest solutions to identified barriers.

Probes:

- Counseling improvements
- Simplified treatment
- Support systems
- Trust-building strategies

Closing

- Summarize key insights (especially around antibiotic use and preferences).
- Ask if participants have additional thoughts on child treatment and medicines.
- Thank participants for their valuable contribution.
