# Supplementary material for: Efficacy of Empirical Antibiotics in Community-Based Management of Uncomplicated Severe Acute Malnutrition in Children: Protocol of a Randomized Controlled Trial
Source: JMIR Res Protoc. 2026 Sep 2;15:e91579. doi: 10.2196/91579 (PMC13583509; doi:10.2196/91579)
Supplement: Supplementary file 3 [file resprot_v15i1e91579_app2.docx]

**In-Depth Interview (IDI) Guides for Healthcare Providers**

**Study Objective:** To explore healthcare provider’s challenges and facilitators in the management of children with uncomplicated Severe Acute Malnutrition (SAM) under the Community-Based Management of Acute Malnutrition (CMAM) Programme in Telangana

**General Instructions for Interviewers**

- Introduce yourself and the purpose of the study.
- Explain confidentiality and voluntary participation.
- Obtain informed consent.
- Seek permission for audio recording.
- Build rapport before beginning sensitive questions.

**IDI GUIDE FOR Accredited Social Health Activist (ASHA)**

**Participant Information**

- Name/Code:
- Age:
- Years of Experience:
- Village/Area:
- Date:
- Interviewer:

**Introduction and Role**

1. Please introduce yourself and share your experience in SSFP programme
2. Please tell me about your role in CMAM at your village level.

(Probes: Daily responsibilities, Role in child nutrition, Role in identifying malnourished children, Involvement in CMAM activities)

3. What do you understand about Severe Acute Malnutrition (SAM)?

(Probes: Signs and symptoms, Causes, Identification methods, Understanding of complications)

4. How do you identify children who may be severely malnourished?

(Probes: Anthropometric assessment, Appetite assessment, Visible signs noticed, Frequency of screening)

5. What role do you play in assessing the health condition of children with uncomplicated SAM under the SSFP programme? (Probes: Identification of danger signs, medical complications, referral decisions, confidence in assessment, challenges during assessment)
6. How do you support immunization services for children with uncomplicated SAM?

(Probes: Tracking missed vaccinations, caregiver compliance, barriers to immunization, coordination with Auxiliary Nurse Midwife (ANM), improving immunization coverage)

7. What is your role in medicine provision and treatment support for children with uncomplicated SAM ?	(Probes: Availability of medicines, antibiotic use, treatment adherence, caregiver response to medicines, challenges in medicine access)
8. How do you ensure the mother gives the antibiotic (Amoxycillin) twice a day for 5 days? What challenges do mothers report regarding the taste or frequency of the medicine?
9. How do you support Anganwadi worker (AWW) in managing children with uncomplicated SAM under the SSFP programme?

(Probes: Joint screening, follow-up visits, family mobilization, coordination challenges, supportive teamwork between ASHA and AWWs)

**Counselling and Community Engagement**

10. How do caregivers usually respond when you inform them that their child is severely malnourished? (Probes: Acceptance or denial, Fear or stigma, Delay in seeking treatment, Family decision-making)
11. What challenges do you face while counselling caregivers? (Probes: Food beliefs and myths, Poverty-related issues, Poor compliance, Family resistance, Caregiver workload, Literacy barriers)
12. What counselling approaches work well in motivating families?

(Probes: Demonstrations, Home visits, Family involvement, Repeated counselling, Use of local examples)

**Treatment Adherence and Follow-Up**

13. What challenges do families face in continuing treatment or follow-up under CMAM?

(Probes: Distance to health facility, Child refusal of therapeutic foods, Caregiver migration, Lack of family support, Competing priorities)

14. What difficulties do you face during home follow-up visits?

(Probes: Household availability, maintaining records, Monitoring compliance, Repeat counselling burden, Community expectations)

15. What factors help children recover successfully under the CMAM programme?

(Probes: Caregiver cooperation, Timely referral, Regular follow-up, Good family practices, Community awareness)

**Coordination and System Support**

16. How do you coordinate with AWWs, ANMs, and Medical Officers (MO) for managing Children with SAM?

(Probes: Referral communication, Joint visits, Reporting systems, Supportive supervision)

17. What support do you receive from the health system for CMAM work?

(Probes: Training, Incentives, Supplies and logistics, Supervisory support, Refresher sessions)

18. What kind of training have you received related to SAM or CMAM?

(Probes: Practical training, Refresher sessions, Confidence in case management, Areas needing improvement)

19. What additional support would help you perform your work better?

(Probes: More training, Better incentives, Community awareness programmes, Improved supplies, Digital tools)

**Recommendations and Closing**

20. In your opinion, what are the major challenges in managing children with uncomplicated SAM under CMAM?
21. What changes would improve the CMAM programme in your area?
22. Is there anything else you would like to share about your experience managing children with uncomplicated SAM?

**IDI GUIDE FOR AWW (ANGANWADI WORKER)**

**Participant Information**

- Name/Code:
- Age:
- Years of Experience:
- Anganwadi Centre:
- Date:
- Interviewer:

**Introduction and Role**

1. Please introduce yourself and share your experience in Community-Based Management of Acute Malnutrition (CMAM) - Supervised Supplementary Feeding Program (SSFP)
2. Please describe your role in child nutrition and growth monitoring under SSFP.

(Probes: Growth monitoring activities, Supplementary nutrition distribution, Identification of malnourished children, Nutrition counselling)

3. What is your understanding of Severe Acute Malnutrition (SAM)?

(Probes: Identification criteria, Common causes, Health risks, Importance of early treatment)

4. What responsibilities do you have under the CMAM programme?

(Probes: Screening, Referral, Counselling, Monitoring progress, Record maintenance)

**Screening and Identification**

5. How do you identify children with uncomplicated SAM at the Anganwadi centre?

(Probes: Anthropometric Assessment, Growth chart interpretation, Frequency of monitoring)

6. What challenges do you face during growth monitoring and screening?

(Probes: Equipment issues, Child cooperation, Caregiver participation, Workload burden, Data recording difficulties)

7. What factors support successful screening and identification?	(Probes: Team support, Community participation, Adequate supplies, Experience and training)
8. What is your experience in conducting appetite tests for children with SAM?

(Probes: Appetite test procedure, child cooperation, caregiver understanding, challenges during testing, interpretation of results)

9. How do you differentiate between complicated and uncomplicated children with SAM during screening and follow-up?

(Probes: Identification criteria, danger signs, medical complications, referral decisions, confidence in classification)

**Nutrition Counselling and Feeding Practices**

10. What feeding practices do you commonly observe among families of children with uncomplicated SAM?

(Probes: Breastfeeding practices, Complementary feeding, Food diversity, Meal frequency, Feeding during illness)

11. What challenges do caregivers face in following nutrition advice?

(Probes: Poverty, Food insecurity, Cultural beliefs, Child feeding difficulties, Family influence)

12. What counselling strategies have been most effective?

(Probes: Recipe demonstrations, Mother meetings, Home counselling, Use of local foods, Repeated reinforcement)

**Treatment Adherence and Follow-Up**

13. What challenges do families face in continuing treatment or follow-up under CMAM?

(Probes: Distance to health facility, Child refusal of therapeutic foods, Caregiver migration, Lack of family support, Competing priorities)

14. What role do you play in providing Take Home Ration (THR) and Balamrutham Plus to children with uncomplicated SAM?

(Probes: Distribution process, availability of THR/Balamrutham Plus, Supply chain gap, caregiver acceptance, sharing within family, compliance issues)

15. What challenges do you face in ensuring regular consumption of THR or Balamrutham Plus among children with uncomplicated SAM?

(Probes: Child refusal, sharing of food, preparation practices, stock availability, caregiver compliance)

16. What difficulties do you face during home follow-up visits?

(Probes: Household availability, maintaining records, Monitoring compliance, Repeat counselling burden, Community expectations)

17. What factors help children recover successfully under the CMAM programme?

(Probes: Caregiver cooperation, Timely referral, Regular follow-up, Good family practices, Community awareness)

**Reporting and record maintenance**

18. How do you record and report information related to children with uncomplicated SAM under the SSFP programme?

(Probes: Register maintenance, Nutrition and health tracking system (NHTS) portal reporting, mobile app usage, data entry process, internet/connectivity issues, reporting burden, accuracy of reporting, technical support received)

19. What challenges do you face while using digital applications or portals for reporting the children with SAM?

(Probes: App usability, login/access issues, lack of training, network problems, duplication of records, time required for data entry, troubleshooting support)

20. What factors help effective reporting and tracking of children with uncomplicated SAM through the NHTS portal or mobile applications?

(Probes: Training support, availability of smartphones/devices, internet access, supervision, simplified reporting systems, coordination with supervisors)

**Programme Implementation Challenges**

21. What operational challenges do you face while implementing CMAM activities?

(Probes: Staff shortages, Documentation burden, Supply interruptions, Time constraints, Coordination issues)

22. What difficulties do families face in attending Anganwadi services regularly?

(Probes: Migration, Distance, Caregiver employment, Family priorities, Social barriers)

23. What factors contribute to successful recovery of children with uncomplicated SAM?

(Probes: Family support, Regular attendance, good counseling, Early identification, Multi-sector coordination)

**Coordination and Support Systems**

24. How do you work with Accredited Social Health Activist **(**ASHAs), Auxiliary Nurse Midwife (ANMs), and Medical Officers in managing children with uncomplicated SAM?

(Probes: Joint follow-up visits, referral coordination, information sharing, counselling support, tracking defaulters)

25. What kind of training have you received related to SAM or CMAM?

(Probes: Practical training, Refresher sessions, Confidence in case management, Areas needing improvement)

26. What support do you receive for CMAM implementation?

(Probes: Training, Monitoring and supervision, Logistics support, Nutrition materials)

27. What factors help successful recovery of children with uncomplicated SAM under SSFP/CMAM?

(Probes: Early identification, regular follow-up, caregiver cooperation, proper THR usage, timely medical referral, teamwork among frontline workers)

28. What improvements would help strengthen CMAM activities?

(Probes: Better infrastructure, Additional staff, Community awareness, Refresher training, Improved supplies.)

**Recommendations and Closing**

29. What are the major barriers in managing children with uncomplicated SAM under CMAM?
30. What changes would improve nutrition outcomes among children with uncomplicated SAM?
31. Is there anything else you would like to share?

**IDI GUIDE FOR ANM**

**Participant Information**

- Name/Code:
- Age:
- Years of Experience:
- Village/Area:
- Date:
- Interviewer:

**Professional Role and Understanding**

1. Please introduce yourself and share your experience in the SSFP programme.
2. Please describe your role & responsibilities in Severe Acute Malnutrition (SAM) child health and nutrition services under SSFP.

(Probes: Growth monitoring, management of childhood illnesses, immunization linkage, referral responsibilities, sub-centre activities)

3. What is your understanding of Severe Acute Malnutrition (SAM) and CMAM?

(Probes: Diagnostic criteria, medical complications, community-based management, referral pathways

**Identification, Assessment and Referral**

4. How are children with SAM usually identified in your area?

(Probes: Community screening process, anthropometric assessment, referral mechanisms, frequency of detection)

5. How do you conduct medical assessment of children with SAM at the sub-centre or during outreach activities?

(Probes: Physical examination, illness identification, appetite assessment, immunization status, danger signs, referral decisions)

6. What common health problems do children with SAM present with?

(Probes: Fever, diarrhoea, pneumonia, feeding difficulties, recurrent infections)

7. How do you differentiate between children with complicated and uncomplicated SAM?

(Probes: Medical complications, appetite test findings, oedema, danger signs, referral criteria, confidence in classification)

8. What challenges do you face in identifying and referring children with SAM?

(Probes: Delayed reporting, inadequate screening skills, lack of equipment, transportation barriers, family refusal

9. What challenges do you face in referring children with SAM for higher medical care based on assessment findings?

(Probes: Caregiver refusal, transport difficulties, delayed referral, coordination with higher facilities, referral follow-up)

10. What factors facilitate timely referral and treatment of children with SAM ?

(Probes: Team coordination, community awareness, supportive supervision, availability of services, caregiver cooperation)

**Clinical, Medicine and Immunization Management**

11. What role do you play in immunization activities for children with SAM?

(Probes: Tracking missed vaccinations, immunization during Village Health Nutrition Days (VHNDs), caregiver awareness, barriers to immunization, follow-up for defaulters)

23. What medicines are commonly provided to children with uncomplicated SAM under SSFP, and what challenges do you face in ensuring proper usage?

(Probes: Antibiotics, deworming, micronutrients, Oral Rehydration Solution/zinc, caregiver understanding, medicine adherence)

24. How is medicine management handled for children with uncomplicated SAM under SSFP at the sub-centre level?

(Probes: Medicine availability, indenting process, stock maintenance, dispensing practices, shortages or delays)

25. What challenges do caregivers face in ensuring adherence and compliance to medicines given for children with uncomplicated SAM?

(Probes: Forgetting doses, side effects, caregiver understanding, child refusal, incomplete treatment, misconceptions about medicines)

26. What challenges do you face while managing children with uncomplicated SAM in the community?

(Probes: Follow-up difficulties, limited treatment options, caregiver non-compliance, heavy workload, incomplete referrals)

27. What factors improve treatment adherence among families of children with uncomplicated SAM?

(Probes: Family counselling, home visits, community support, trust in health workers, repeated follow-up)

**Reporting, Programme Functioning and Coordination**

28. How do you record and report the information related to children with uncomplicated SAM under SSFP? How is your experience with reporting and documentation related to children with SAM either using register or digital systems?

(Probes: Register maintenance, Mother and Child Health Kit (MCH Kit) reporting, digital reporting systems, reporting accuracy, connectivity issues, data entry burden)

29. What role do you play in organizing and managing VHNDs for children with uncomplicated SAM

(Probes: Growth monitoring, counselling, immunization, referral activities, medicine distribution, operational challenges)

30. How effectively does coordination occur between frontline workers and health facilities?

(Probes: Communication systems, reporting flow, joint planning, referral tracking)

31. How do you coordinate with ASHAs, Mid-level Health Providers (MLHPs) Medical Officers in the management of children with uncomplicated SAM?

(Probes: Referral communication, joint follow-up, supervision, information sharing, case tracking, teamwork challenges)

32. What operational difficulties affect CMAM/SSFP implementation?

(Probes: Staff shortage, training gaps, supply chain issues, documentation burden, transportation challenges)

33. What support systems help CMAM/SSFP implementation function well?

(Probes: Supportive supervision, refresher training, medicine availability, logistics support, community participation)

**Capacity Building and Recommendations**

34. What kind of training have you received related to SAM or CMAM?

(Probes: Practical training, refresher sessions, confidence in case management, areas needing improvement)

35. What additional support would help improve management of children with uncomplicated SAM?

(Probes: Logistics support, digital reporting support, additional workforce, better coordination, regular supplies)

36. What changes would strengthen CMAM/SSFP implementation in Telangana?

**Closing**

37. Is there anything else you would like to share regarding your experiences with SAM management?

**IDI GUIDE FOR MEDICAL OFFICER (MO)**

**Participant Information**

- Name/Code:
- Qualification:
- Years of Experience:
- Facility:
- Date:
- Interviewer:

**Professional Experience and Understanding**

1. Can you please introduce yourself and share your experience in the CMAM/SSFP programme?
2. Please describe your role in managing children with uncomplicated SAM under CMAM/SSFP.
   (Probes: Clinical responsibilities, supervision of frontline workers, referral oversight, monitoring outcomes, medicine management)
3. How are children with SAM typically managed at your facility?
   (Probes: Screening protocols, clinical assessment, identification of complicated and uncomplicated SAM, referral decisions, follow-up process)

**Clinical Management and Antibiotic Practices**

4. What clinical challenges do you commonly encounter while managing children with SAM?
   (Probes: Co-morbidities, severe infections, late presentation, recurrent illness, caregiver adherence, referral delays)
5. What challenges exist in identifying complicated versus uncomplicated SAM cases?
   (Probes: Diagnostic limitations, staff skills, appetite assessment, resource constraints, monitoring difficulties)
6. What is your perspective on the use of antibiotics in the management of children with uncomplicated SAM under CMAM/SSFP?

(Probes: Routine antibiotic prescription, indications for use, complicated versus uncomplicated SAM, commonly prescribed antibiotics, adherence to guidelines)

7. In your opinion, how necessary are antibiotics for children with uncomplicated SAM managed at the community level? (Probes: Benefits of antibiotics, situations where antibiotics may not be required, clinical judgement, caregiver expectations, treatment outcomes)
8. What challenges do you face regarding antibiotic use among children with uncomplicated SAM? (Probes: Irrational antibiotic use, incomplete treatment, caregiver compliance, overuse of antibiotics, medicine availability, follow-up issues)
9. What are your views on antibiotic resistance in relation to SAM management?
   (Probes: Antimicrobial resistance, inappropriate prescriptions, repeated antibiotic exposure, community-level resistance concerns, antibiotic stewardship)
10. What is your opinion on using a single dose of Azithromycin compared to a one-week course of Amoxicillin for children with uncomplicated SAM under CMAM/SSFP?
    (Probes: Effectiveness, feasibility, caregiver compliance, ease of administration, side effects, treatment adherence, antimicrobial resistance, suitability for community-based management)
11. What challenges do caregivers face in accessing and continuing treatment for children with uncomplicated SAM (Probes: Financial barriers, transport issues, medicine adherence, family support, awareness levels)
12. What factors support successful management and recovery of children with uncomplicated SAM?
    (Probes: Early detection, strong referral systems, caregiver compliance, availability of medicines and supplies, staff coordination, follow-up systems)

**Health System and Programme Implementation**

13. What operational challenges affect CMAM/SSFP implementation at the facility level?
    (Probes: Human resource constraints, supply chain interruptions, documentation burden, inadequate infrastructure, patient load)
14. How effective is coordination between frontline workers and health facilities in managing children with uncomplicated SAM?

(Probes: Referral communication, follow-up tracking, supportive supervision, continuity of care, joint review meetings)

15. What is your experience with reporting and monitoring systems related to children with uncomplicated SAM? (Probes: Data reporting, digital systems, reporting burden, monitoring indicators, data quality, technical support)
16. What support mechanisms would help improve CMAM/SSFP implementation?
    (Probes: Administrative support, refresher training, workforce strengthening, logistics support, uninterrupted supplies)

**Recommendations and Closing**

17. In your opinion, what are the major barriers to effective CMAM/SSFP implementation?
18. What programme or policy changes would improve management and recovery of children with SAM in Telangana?
19. Is there anything else you would like to share about CMAM/SSFP implementation or management of children with uncomplicated SAM?

**IDI GUIDE FOR PEDIATRICIAN**

**Participant Information**

- Name/Code:
- Qualification:
- Years of Experience:
- Institution:
- Date:
- Interviewer:

**Clinical Experience and Understanding**

1. Can you please introduce yourself and share your experience in treating children with uncomplicated SAM under CMAM/SSFP?
2. Please describe your experience in treating children with Severe Acute Malnutrition.
   (Probes: Case load, common presentations, referral patterns, clinical outcomes)
3. What is your perspective on the CMAM/SSFP approach for managing children with uncomplicated SAM? (Probes: Advantages, limitations, community-based care effectiveness, referral linkages)
4. How are children with SAM typically managed at your institution?
   (Probes: Assessment protocols, identification of complicated and uncomplicated SAM, admission criteria, outpatient versus inpatient care, follow-up mechanisms)

**Clinical and Antibiotic Management Challenges**

5. What major clinical challenges do you encounter while managing children with uncomplicated/ complicated SAM?
   (Probes: Severe infections, delayed presentation, feeding complications, relapse cases, mortality risks)
6. What is your perspective on the use of antibiotics in the management of children with uncomplicated SAM under CMAM/SSFP?
   (Probes: Routine antibiotic prescription, indications for use, complicated versus uncomplicated SAM, commonly prescribed antibiotics, adherence to guidelines)
7. In your opinion, how necessary are antibiotics for children with uncomplicated SAM managed at the community level?
   (Probes: Clinical benefits, situations where antibiotics may not be required, evidence-based practice, outpatient management, risk of untreated infections)
8. What challenges do you observe regarding antibiotic usage among children with uncomplicated SAM?
   (Probes: Irrational antibiotic use, incomplete treatment, caregiver compliance, self-medication, overuse of antibiotics, follow-up challenges)
9. What are your views on antibiotic resistance in relation to SAM management?
   (Probes: Antimicrobial resistance concerns, repeated antibiotic exposure, inappropriate prescriptions, resistance patterns, impact on treatment outcomes)
10. What is your opinion on using a single dose of Azithromycin compared to a one-week course of Amoxicillin for children with uncomplicated SAM under CMAM/SSFP?
    (Probes: Effectiveness, feasibility, caregiver compliance, ease of administration, side effects, treatment adherence, relapse risk, antimicrobial resistance)
11. What challenges affect continuity of care and treatment adherence among children with uncomplicated SAM after discharge or referral?
    (Probes: Follow-up adherence, caregiver understanding, weak referral systems, medicine compliance, repeated visits, financial burden)
12. What factors contribute to better treatment outcomes among children with uncomplicated SAM?
    (Probes: Early intervention, appropriate feeding practices, caregiver engagement, strong follow-up systems, timely referral)

**Health System and Programme Implementation**

13. From your perspective, what are the major strengths and weaknesses of the CMAM/SSFP programme?
    (Probes: Community reach, early identification, hospital burden reduction, human resources, referral coordination, supply issues)
14. How effective is coordination between community workers and higher health facilities?
    (Probes: Referral quality, communication systems, follow-up feedback, continuity of care)
15. What system-level barriers affect effective SAM management in Telangana?
    (Probes: Workforce shortages, infrastructure gaps, monitoring systems, data quality, funding limitations)
16. What recommendations would you suggest to strengthen CMAM/SSFP implementation and rational antibiotic use?
    (Probes: Antibiotic stewardship, policy changes, provider training, improved referral systems, strengthening frontline workforce, better nutrition support)

**Closing**

17. Is there anything else you would like to share regarding SAM management or CMAM/SSFP implementation?
